# Elucidating Turnover Pathways of Bioactive Small Molecules by Isotopomer Analysis: The Persistent Organic Pollutant DDT

**DOI:** 10.1371/journal.pone.0110648

**Published:** 2014-10-28

**Authors:** Ina Ehlers, Tatiana R. Betson, Walter Vetter, Jürgen Schleucher

**Affiliations:** 1 Department of Medical Biochemistry & Biophysics, Umeå University, Umeå, Sweden; 2 Department of Food Chemistry, University of Hohenheim, Stuttgart, Germany; Martin-Luther-Universität Halle-Wittenberg, Germany

## Abstract

The persistent organic pollutant DDT (1,1,1-trichloro-2,2-bis(4-chlorophenyl)ethane) is still indispensable in the fight against malaria, although DDT and related compounds pose toxicological hazards. Technical DDT contains the dichloro congener DDD (1-chloro-4-[2,2-dichloro-1-(4-chlorophenyl)ethyl]benzene) as by-product, but DDD is also formed by reductive degradation of DDT in the environment. To differentiate between DDD formation pathways, we applied deuterium NMR spectroscopy to measure intramolecular deuterium distributions (^2^H isotopomer abundances) of DDT and DDD. DDD formed in the technical DDT synthesis was strongly deuterium-enriched at one intramolecular position, which we traced back to ^2^H/^1^H fractionation of a chlorination step in the technical synthesis. In contrast, DDD formed by reductive degradation was strongly depleted at the same position, which was due to the incorporation of ^2^H-depleted hydride equivalents during reductive degradation. Thus, intramolecular isotope distributions give mechanistic information on reaction pathways, and explain a puzzling difference in the whole-molecule ^2^H/^1^H ratio between DDT and DDD. In general, our results highlight that intramolecular isotope distributions are essential to interpret whole-molecule isotope ratios. Intramolecular isotope information allows distinguishing pathways of DDD formation, which is important to identify polluters or to assess DDT turnover in the environment. Because intramolecular isotope data directly reflect isotope fractionation of individual chemical reactions, they are broadly applicable to elucidate transformation pathways of small bioactive molecules in chemistry, physiology and environmental science.

## Introduction

The chloropesticide DDT (1,1,1-trichloro-2,2-bis(4-chlorophenyl)ethane) is one of the most controversial chemicals developed in the twentieth century. Its application as the first effective pesticide to combat malaria has saved the lives of millions of people. This success triggered its general use as a pesticide on a million-ton scale in agriculture and by households worldwide. Due to its detrimental properties (lipophilicity and persistency), the use of DDT has led to its ubiquitous occurrence in the environment even in remote areas where it has never been used [1], [2]. In addition, serious toxicological effects such as thinning of egg shells of birds in the Baltic region have been linked to DDT [3]. As a consequence, DDT has strongly influenced the public perception and regulation of man-made chemicals. Although the use of DDT has been drastically reduced for decades, it is still abundant in the environment and is still having detrimental effects. For this reason, DDT has been classified as a persistent organic pollutant (POP) by the Stockholm Convention, with the purpose of phasing out its use globally. However, it is still used against vector-borne diseases [4], primarily malaria transmitted by *Anopheles* mosquitos.

**Figure 1:**
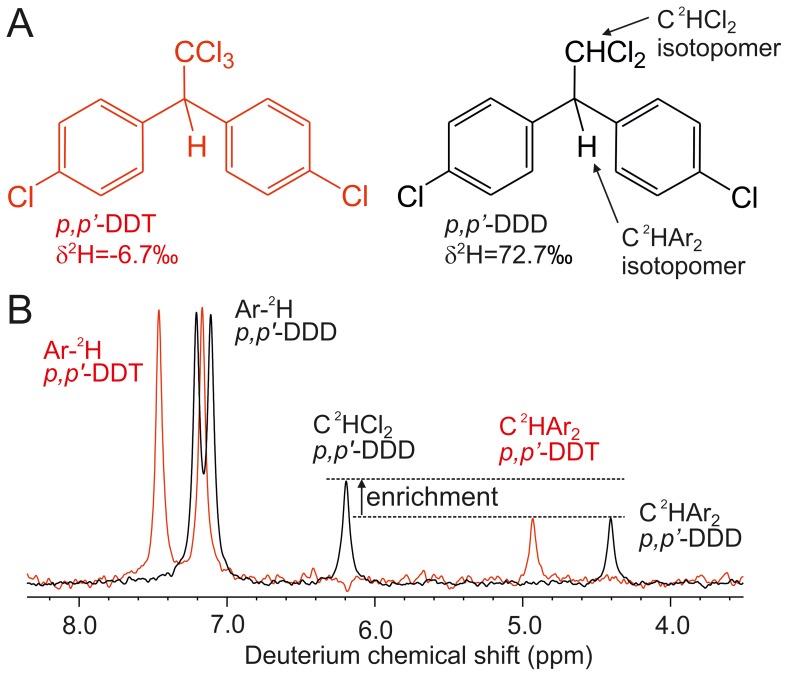
Comparison of *p, p′*-DDT with its congener *p, p′*-DDD. (A) Chemical structures and whole-molecule δ^2^H values. (B) Overlay of deuterium NMR spectra of reference samples of *p, p*′-DDD (black, reference 1) and *p, p′*-DDT (red). Integrals of the signals are proportional to the abundance of the respective ^2^H isotopomers. Ar-^2^H denotes isotopomers carrying ^2^H in the aromatic moieties of the respective compound. Positions of signals (“chemical shifts”) differ between DDT and DDD because they reflect the stereoelectronic properties of each molecule; these chemical shift differences do not influence the integration of signals.

Since the discovery of the persistence of DDT, numerous studies have addressed environmental and human health hazards of DDT and its congeners [5], [6]. Recent studies have detected *p, p′*-DDE (1,1-bis-(4-chlorophenyl)-2,2-dichloroethene) in human blood serum [6], even in countries that stopped DDT usage decades ago, and link high *p, p′*-DDE blood serum levels with prevalent hypertension [7] and an increased risk of Alzheimer disease [8]. Technical DDT is a mixture of compounds, the major components being the *p, p′* and *o, p′* isomers of DDT and some of the minor components being the *p, p′* and *o, p′* isomers of DDD (1-chloro-4-[2,2-dichloro-1-(4-chlorophenyl)ethyl]benzene) [9] (Fig. 1). DDD not only occurs as a by-product of technical DDT production but has itself been used as a pesticide, and is formed in the environment by hydrodechlorination of DDT [9]–[11]. DDD is of particular ecotoxicological relevance because it accumulates in the environment and has been implicated as a possible endocrine disruptor [5], [12]. As for POPs in general [13], tracing DDT in the environment is critical to understand its transformation processes, to judge environmental and human health risks, and to develop remediation strategies.

Increasingly, stable heavy isotopes, which occur naturally in the environment with varying abundance, are used to trace compounds [14]. In most applications, the abundance ratio between a heavy isotope and a light isotope of a chemical element is measured by isotope-ratio mass spectrometry (IRMS). Variation observed in isotope ratios is usually expressed as δ value (in ‰), the abundance deviation of the respective heavy isotope relative to a standard with known isotope ratio [15]. However, IRMS only allows measurement of the average isotope ratio of a whole molecule, because any molecule to be studied must be converted into defined gases for IRMS analysis. It is known, though, that the abundance of heavy isotopes varies among intramolecular groups of non-symmetric molecules [16], but the consequences of this intramolecular variation cannot be assessed by whole-molecule δ measurements. A molecule carrying a specific isotope at a specified intramolecular position is called an isotopomer. Intramolecular isotope variation therefore means that the isotopomers of non-symmetric molecules differ in their abundances. This variation in abundance is mainly caused by kinetic isotope effects during chemical reactions. Thus, isotopomer abundances are directly related to reaction mechanisms and turnover along chemical pathways [17]. As has been observed for several types of compounds, kinetic protium/deuterium (^1^H/^2^H) isotope effects can easily double the abundance of particular ^2^H isotopomers [18]–[20]. Relative abundances of ^2^H isotopomers can be measured by deuterium NMR, because each ^2^H isotopomer creates one signal in the deuterium NMR spectrum. If the signals can be resolved and using suitable experimental conditions, the integrals of the deuterium NMR spectrum directly reflect relative isotopomer abundances [18], [21], [22]. Furthermore, when isotopomer abundance can be linked to defined chemical steps, isotopomer variation yields mechanistic information on reaction pathways. Here we use deuterium NMR to identify ^2^H fractionations due to individual reactions during DDD formation, as basis to distinguish DDD formed in technical DDT production from DDD formed by hydrodechlorination of DDT in the environment.

## Results & Discussion

Reference samples of *p, p′*-DDT and *p, p′*-DDD have been found to differ by as much as 79‰ in δ^2^H [23] (Fig. 1A). Variation in this range would normally rule out a common source of the compounds. We measured deuterium NMR spectra of the same *p, p′*-DDT and *p, p′*-DDD reference samples, which show indistinguishable abundance patterns for the C^2^HAr_2_ and Ar-^2^H isotopomers (Fig. 1B). In contrast, the C^2^HCl_2_ isotopomer in DDD was nearly twice as abundant as the C^2^HAr_2_ isotopomer (Table 1, *p, p′*-DDD reference sample 1). By combining relative isotopomer abundances from NMR spectra with the δ^2^H values of the compounds [23], we calculated how much the high abundance of the C^2^HCl_2_ isotopomer influenced the δ^2^H of DDD. This isotope balance calculation (see Materials and Methods) shows that the high abundance of the C^2^HCl_2_ isotopomer (δ^2^H = 739‰) completely explains the 79‰ difference in whole-molecule δ^2^H between *p, p′*-DDT and *p, p′*-DDD. Thus, the molecular part that is common to DDD and DDT (CHAr_2_ fragment) showed indistinguishable δ^2^H values (Table 1). Consequently, the isotopomer analysis demonstrates that both compounds can have a common source.

**Table 1:** Deuterium abundance data of DDT and related compounds.

Sample		C^2^HCl_2_ isotopomerabundance^[a]^	δ^2^H, ‰	
			Whole molecule^[b]^	CHAr_2_ fragment^[c]^
*p, p′*-DDT reference		–	−6.7±3.4	−6.7±3.4
*p, p′*-DDD reference 1		1.77±0.10	72.7±7.0	−1.3±11.4
*p, p′*-DDD reference 2		1.80±0.04	–	–
*p, p′*-DDD reference 3		1.76±0.11	–	–
*o, p′*-DDD reference		1.89±0.09	74.8±7.6	−6.8±10.2
Commercial CHCl_2_CHO		1.27±0.02	–	–
*p, p′*-DDD in technical DDT			–	–
replicate 1	2.17±0.44		–	–
replicate 2	1.78±0.26		–	–
*p, p′*-DDD from DDT hydrodechlorination:				
Experiment 1	0.69±0.05		–	–
Experiment 2	0.62±0.07		–	–
Experiment 3	0.58±0.09		–	–

[a] Relative to C^2^HR_2_ = 1.

[b] from reference [23].

[c] See Materials and Methods for calculations.

Measurements on two further independent samples of *p, p′*-DDD (reference 2 and 3 in Table 1) gave indistinguishable results. Furthermore, analogous experiments on reference material of the *o, p′* isomer of DDD showed that the C^2^HCl_2_ isotopomer was again almost twice as abundant as the C^2^HAr_2_ isotopomer (Table 1). These results suggest that the C^2^HCl_2_ isotopomer of synthetic DDD generally has a very high abundance, which significantly shifts the whole-moleculeδ^2^H value.

**Figure 2:**
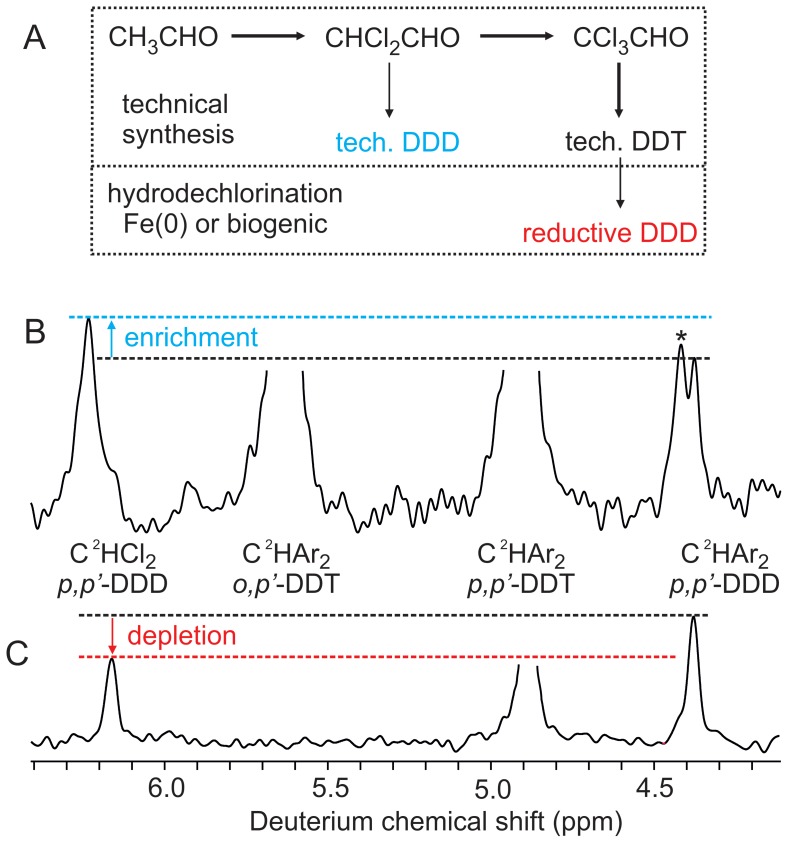
Pathways of DDD formation and their ^2^H fractionation. (A) Technical synthesis of DDT, and pathways of formation of DDD. (B) Deuterium NMR spectrum of technical DDT (replicate 1). (C) Deuterium NMR spectrum of a reaction mixture containing DDD formed by hydrodechlorination (experiment 1). Blue and red lines qualitatively illustrate enrichment or depletion of the C^2^HCl_2_ isotopomer in technical and reductive DDD, respectively. To take differing linewidths and overlap into account, numerical values for isotopomer abundances were obtained by deconvolution of the signals. Note that signal positions differ slightly between samples, because of differing analyte concentrations. The asterisk denotes an unknown impurity in technical DDT.

To apply isotopomer analysis to DDT as used in practice, we recorded deuterium NMR spectra of technical DDT (Fig. 2B) to analyze the by-product *p, p′*-DDD. Technical DDT is synthesized by reacting CCl_3_CHO with chlorobenzene (Fig. 2A). The technical product contains only 4% DDD, and the amount of DDD in the technical DDT sample was only approximately 15 µmol. Deconvolution allowed quantifying the signals originating from *p, p′*-DDD, although the signal of the C^2^HCl_2_ isotopomer partly overlapped with the signal of an unknown impurity. Measurements on two replicate NMR samples of the same technical DDT gave similar results (Table 1), which showed that its C^2^HCl_2_ isotopomer was about twice as abundant as the C^2^HAr_2_ isotopomer. This indicates that acceptable signal-to-noise ratios can be obtained using sample amounts of 15 µmol, using a cryogenically cooled probe [24]. The synthesis of CCl_3_CHO proceeds via chlorination of CH_3_CHO. Incomplete chlorination gives rise to residual CHCl_2_CHO from which the corresponding DDD isomers are formed. Chlorination of carbonyl compounds is associated with a kinetic ^1^H/^2^H isotope effect of up to seven [25], which is close to the maximum classical kinetic ^1^H/^2^H isotope effect for C–H bond breakage [26]. According to the theory of isotope effects [26], chlorination of CH_3_CHO will - with increasing turnover - progressively enrich deuterium in residual CHCl_2_ groups. Supporting this explanation, a commercial CHCl_2_CHO product showed a 1.27-fold enrichment of the C^2^HCl_2_ isotopomer compared to the C^2^HO isotopomer (Table 1). Thus, we conclude that the high abundance of the C^2^HCl_2_ isotopomer of DDD is the result of the ^1^H/^2^H isotope effect during the chlorination of CH_3_CHO. The original enigma of how *p, p′*-DDD and *p, p′*-DDT can have a difference in δ^2^H of 79‰ can be attributed to the isotope effect during synthesis of CCl_3_CHO.

DDT is known to be degraded into DDD in the environment through hydrodechlorination under anaerobic conditions [10]. In this reaction, a chlorine atom in the CCl_3_ moiety of DDT is replaced by a hydride equivalent to form the CHCl_2_ group of DDD. The hydride equivalents are thought to be derived from the biological cofactors FADH_2_ or reduced Fe porphyrins [10]. An analogous hydrodechlorination reaction using Fe(0) has been proposed for remediation of DDT contamination [11]. Thus, we experimentally produced DDD by hydrodechlorination of *p, p′*-DDT with Fe(0). The deuterium NMR spectrum of the resulting reaction mixture (Fig. 2C) shows that the *p, p′*-DDD formed by hydrodechlorination was approximately 30% depleted in the C^2^HCl_2_ isotopomer. To test the reproducibility of the method, we carried out triplicate experiments (see Material S1), which gave statistically indistinguishable results with an average abundance of the C^2^HCl_2_ isotopomer of 0.63±0.05, relative to the C^2^HPh_2_ isotopomer. The strong ^2^H depletion of the CHCl_2_ group formed by hydrodechlorination is for a fundamental biophysical reason. Because ^1^H^2^HO is a weaker acid than H_2_O, the hydronium ions in water are ^2^H-depleted by approximately −700 ‰ [27], [28]. When these ions enter reduction reactions leading to abiogenic or biogenic hydride equivalents, the resulting hydride equivalents become accordingly ^2^H-depleted. The resulting depletion, of the order of −300‰, has been observed in hydride-derived isotopomers of biochemical metabolites [17]. Thus, a strong ^2^H depletion of the CHCl_2_ group, similar to our Fe(0) reduction experiment, can be expected for DDD formed by hydrodechlorination of DDT in the environment. This depletion can serve to identify DDD formed in situ, either as result of natural degradation, or of remediation efforts, and can therefore be used to monitor breakdown of DDT.

In summary, DDD from technical synthesis is characterized by an increased abundance of the C^2^HCl_2_ isotopomer, while DDD formed by reductive dechlorination is depleted in the C^2^HCl_2_ isotopomer, and both isotopomer fractionations are inherent properties of the respective pathway of DDD generation. In light of the opposite deuterium fractionations, technical DDD and DDD formed by hydrodechlorination should generally differ by approximately 1,000‰ in the abundance of the C^2^HCl_2_ isotopomer (Table 1). This isotopomer difference can be used to estimate fractional contributions of both pathways to environmental samples of DDD. Unfortunately, the sample amount currently required for ^2^H isotopomer measurements by NMR (approximately 10 mg per congener, see above) restricts the application of this approach to highly contaminated sites where large quantities of DDT congeners can be isolated. However, the C^2^HCl_2_ isotopomer signatures of technical and environmental DDD formation respectively induce more positive or negative whole-molecule δ^2^H values of DDD, as compared to DDT. Compound-specific IRMS methods for δ^2^H measurements for polyhalogenated compounds [29], [30] have recently been introduced; therefore comparison of δ^2^H values of DDD and DDT may allow tracing sources of DDD to technical synthesis or breakdown of DDT in the environment.

## Conclusion

Our D isotopomer measurements represent a striking demonstration that individual ^2^H isotopomer abundances can have a decisive effect on whole-molecule δ^2^H values. Hence isotopomer abundances give essential insights into causes of variation in δ^2^H, and must be taken into account in interpretations of whole-molecule isotope ratios, even when structural differences between related compounds appear to be minor. For DDT, sources of DDD may be traced based on isotopomer results. In general, isotopomer analysis of POPs can be applied: (1) to identify the responsible polluter; (2) to understand POP transformation processes in the environment; and (3) to gauge the efficacy of remediation approaches.

Isotope fractionation of chemical reactions directly affects isotopomer abundances, which in turn affects whole-molecule isotope ratios. However, without prior knowledge a change in a whole-molecule isotope ratio cannot be related to fractionation by a chemical reaction. Therefore isotopomer measurements are particularly valuable for deducing mechanistic detail of reaction pathways. The availability of cryogenically cooled probes has reduced the sample amount needed for isotopomer analysis to only micromoles, which enables applications in fields ranging from organic and pharmaceutical chemistry, to metabolic studies, and environmental science.

## Materials and Methods

### Sources of compounds

See Material S1.

### Deuterium NMR

Several conditions must be fulfilled for isotopomer measurements by NMR. First, line widths must be minimized to resolve the signals and to optimize sensitivity. To achieve this goal, we used low-viscosity solvents C_6_F_6_ or hexane/C_6_F_6_ and elevated measurement temperatures around 50°C [21]. Second, the NMR signals must have pure Lorentzian lineshapes, so that their integrals can be determined by lineshape fitting. This goal was achieved by careful homogenizing the magnetic field of the NMR spectrometer (“shimming”). Third, because many individual spectra (“scans”) are added to obtain each NMR spectrum, the influence of the previous scan on the deuterium nuclei must have decayed when the next scan is acquired. To achieve this goal, we determined deuterium relaxation times (T_1_) of all compounds using an inversion-recovery pulse sequence, and acquired spectra with appropriate recycle times between scans (6 T_1_) to ensure complete relaxation. A DRX600 spectrometer (Bruker, Karlsruhe, Germany) equipped with a 5-mm broadband probe and a ^19^F lock device was used to record ^2^H NMR spectra of DDT and DDD reference materials; C_6_F_6_ was used for field-frequency locking. Spectra of dichloroacetaldehyde diethyl acetal were acquired on a DRX500 spectrometer in unlocked mode using a 10-mm broadband probe. Spectra of technical DDT and of the hydrodechlorination reaction mixture were obtained on an Avance III 850 spectrometer equipped with a cryogenic probe optimized for deuterium detection and equipped with ^19^F lock. Parameter settings for all samples are given in Material S1. All spectra were recorded with proton decoupling. Spectra were processed with exponential line-broadening, and signal integrals were obtained by deconvolution in the program topspin (version 3.0; Bruker). Typically, five spectra were recorded. The standard deviation of the C^2^HCl_2_/C^2^HR_2_ abundance ratio among replicate spectra was used to estimate the overall precision of our measurements. For three independent hydrodechlorination experiments, the standard deviations of replicate spectra were 0.05, 0.07 and 0.09, respectively. The experiments led to results which did not significantly differ from each other (ANOVA), and the standard deviation among the experiments was 0.05, indicating that the standard deviation among spectra is a realistic estimate of the overall precision (Table 1).

### DDT hydrodechlorination

*p, p′*-DDT was reduced with Fe(0) under anaerobic conditions following Sayles et al. [11]. A 1-L flask was filled with 1 L 20 mM MOPS buffer (pH 7, deoxygenated by purging with N_2_); 15 g Fe(0) powder and 60 mg *p, p′*-DDT (dissolved in 20 mL acetone) were added. The flask was sealed and mixed by rotation. After 18 h at room temperature, the reaction mixture was extracted consecutively with *n*-hexane and chloroform. The organic phases were dried with Na_2_SO_4_, the solvent was removed under vacuum, and the resulting product was used for NMR measurements. Analysis of the product using ^1^H NMR showed that the main components of the product were un-reacted *p, p′*-DDT and approximately 30% *p, p′*-DDD.

### Isotope balance calculations

Deuterium NMR spectra yield relative abundances of ^2^H isotopomers, but position-specific ^2^H/^1^H ratios cannot be derived from NMR alone. When the whole-molecule ^2^H/^1^H ratio is available and if all the signals in the ^2^H NMR spectrum can be integrated, position-specific ^2^H/^1^H ratios can be calculated according to the equation 1,
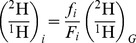
(1)where (^2^H/^1^H)*_i_* and (^2^H/^1^H)*_G_* are the isotope ratios of isotopomer *i* and of the whole molecule, respectively. *f_i_* is the experimentally determined molar fraction of isotopomer *i*, that is, the integral of its NMR signal compared to the integral of all isotopomers of the molecule. *F_i_* is the statistical molar fraction [31], that is, the stoichiometric fraction hydrogen in position *i* according to the molecular formula, and the term *f_i_*/*F_i_* represents the abundance of the ^2^H isotopomer *i*, relative to the ^2^H/^1^H ratio of the whole molecule. Note that the isotope ratios (^2^H/^1^H)*_i_* and (^2^H/^1^H)*_G_* must be expressed as true ratios, i.e. δ^2^H = 72.7‰ for *p, p′*-DDD (Table 1) must be converted to (^2^H/^1^H)*_G_* = 1.0727, i.e. the DDD sample has a 1.0727-fold higher ^2^H/^1^H ratio than VSMOW. For example, the C^2^HCl_2_ isotopomer of *p, p′*-DDD contributed fraction *f*_C2HCl2_ = 0.1621 to the integral of the ^2^H NMR spectrum, while *F*_C2HCl2_ is 0.1 (1 hydrogen out of 10 in the molecule). Therefore the ^2^H/^1^H ratio of the CHCl2 group is (^2^H/^1^H) _C2HCl2_ = 1.621 × 1.0727 = 1.739 and means that this isotopomer has an abundance corresponding to a position-specific δ^2^H of 739‰. An analogous calculation for the rest of the molecule–- the CHAr_2_ fragment (*f*_rest_ = 1 - *f*_C2HCl2_ = 0.8379, *F*_rest_ = 0.9) which is common to *p, p′*-DDT and *p, p′*-DDD–- yields a ^2^H/^1^H ratio of 0.9987, corresponding to a δ^2^H of −1.3‰ for this fragment. The 74‰ difference between this number and the whole-molecule δ^2^H shows how much the whole-molecule δ^2^H is influenced by the high abundance of the C^2^HCl_2_ isotopomer. In the isotope balance results in Table 1, error estimates reflect the combined uncertainties of the whole-molecule δ^2^H measurements and of NMR-derived ^2^H isotopomer distributions. For the DDD component of technical DDT, this calculation is not possible because the aromatic signals of all congeners overlap.

## Supporting Information

Material S1 **List of used spectrometers, measuring parameters and raw data.**
(XLS)
